# Improving and re-developing the Clerical Resolution Online Widget (CROW): open-source software for clerical review

**DOI:** 10.23889/ijpds.v9i5.2579

**Published:** 2024-09-18

**Authors:** Hannah O'Dair, Sarah Cummins, Rachel Huck

**Affiliations:** 1 Office for National Statistics

## Introduction

Due to the availability of data and the speed of delivery required, NHS number (Health ID) was used during the COVID-19 pandemic to join different health datasets to census 2021. This research sought to assess the quality of linkage done by NHS number, between COVID-19 vaccinations data and census 2021, by comparing to the quality that could be achieved with deterministic linkage using Personal Identifiable Information (PII). Specifically, we wanted to assure against concern that unlinked records may have been misclassified as unvaccinated due to inaccurate recording of NHS number at time of vaccination.

## Approach

We used deterministic (rules-based) linkage to identify extra links that were not made on NHS number. We also investigated how many links were incongruent between NHS number linkage and PII deterministic linkage.

## Results

We used deterministic (rules-based) linkage to identify extra links that were not made on NHS number. We also investigated how many links were incongruent between NHS number linkage and PII deterministic linkage.

## Conclusion

We were able to assure that the NHS number linkage used to produce COVID-19 vaccinations statistics, was valid. Whilst 1,833,626 more links were made, the findings resulting from the linked data were unchanged.

## Implications

Whilst this research demonstrates the utility of NHS number-based linkage, acquisition and use of PII on health datasets can increase the link rate and plays a vital role in estimating linkage error.

